# Biased visuospatial perception in complex regional pain syndrome

**DOI:** 10.1038/s41598-017-10077-8

**Published:** 2017-08-29

**Authors:** Lieve Filbrich, Andrea Alamia, Charlotte Verfaille, Anne Berquin, Olivier Barbier, Xavier Libouton, Virginie Fraselle, Dominique Mouraux, Valéry Legrain

**Affiliations:** 1 Institute of Neuroscience, Université catholique de Louvain, Brussels, Belgium; 2 University Hospital Saint-Luc, Brussels, Belgium; 3 Institute of Experimental and Clinical Research, Université catholique de Louvain, Brussels, Belgium; 4 Faculty of Motor Sciences, Université catholique de Louvain, Brussels, Belgium; 5 Faculty of Motor Sciences, Université libre de Bruxelles, Brussels, Belgium; 6 University Hospital Erasme, Brussels, Belgium

## Abstract

Complex regional pain syndrome (CRPS) is a chronic pain condition associating sensory, motor, trophic and autonomic symptoms in one limb. Cognitive difficulties have also been reported, affecting the patients’ ability to mentally represent, perceive and use their affected limb. However, the nature of these deficits is still a matter of debate. Recent studies suggest that cognitive deficits are limited to body-related information and body perception, while not extending to external space. Here we challenge that statement, by using temporal order judgment (TOJ) tasks with tactile (i.e. body) or visual (i.e. extra-body) stimuli in patients with upper-limb CRPS. TOJ tasks allow characterizing cognitive biases to the advantage of one of the two sides of space. While the tactile TOJ tasks did not show any significant results, significant cognitive biases were observed in the visual TOJ tasks, affecting mostly the perception of visual stimuli occurring in the immediate vicinity of the affected limb. Our results clearly demonstrate the presence of visuospatial deficits in CRPS, corroborating the cortical contribution to the CRPS pathophysiology, and supporting the utility of developing rehabilitation techniques modifying visuospatial abilities to treat chronic pain.

## Introduction

Complex regional pain syndrome (CRPS) is a chronic pain condition associating sensory, motor, trophic and autonomic symptoms in one limb (for a review see ref. 1). The pathophysiology of CRPS is complex and difficult to understand as it can involve different mechanisms such as neurogenic inflammation, vasomotor dysfunction as well as structural and functional changes at the cortical level^2, 3^. Probably as a consequence of these cortical changes, unilateral cognitive symptoms have also been described in CRPS, affecting the patients’ abilities to represent, use and perceive their affected limb (for reviews see e.g. refs 4–6). Although there is no clear definition for the concept of body representation, as it encompasses different notions which are often used in different manners and sometimes with opposite meanings by different authors^7^, disturbances in mentally representing their affected limb have been increasingly recognized as being part of the symptomatology in an important subset of CRPS patients^5^. For instance, patients regularly report feelings of disownership over the affected limb^8–11^, as well as distortions in representing its size, shape^11–13^ and position^14^. Furthermore, altered laterality recognition for the affected limb has been repeatedly demonstrated^15–18^. At the motor level, patients described struggling in order to voluntarily move their limb, with movements that are difficult to initiate (hypokinesia) and slower (bradykinesia)^8, 9, 11^. Because there are similarities of the aforementioned symptoms with those reported in hemispatial neglect, the cognitive symptoms observed in CRPS have often been considered as “neglect-like” symptoms^1, 8, 9^ (however, see refs 4, 19 for a discussion). Hemispatial neglect is a cognitive disorder consecutive to lesions in one cortical hemisphere, characterized by an inability to orient to, explore and report sensory events from the side of space controlateral to the damaged hemisphere, which cannot be attributed to peripheral sensory or motor loss^20–22^. Despite the fact the hemispatial neglect can affect different sensory modalities, as well as representational^23^ and motor processing^24, 25^, patients’ symtoms are often, if not almost exclusively, characterized by tests investigating their perceptual abilities in the visuospatial domain, that is, their abilities to perceive and locate stimuli in extra-body space^26–28^. However, neuropsychological tests classically used to investigate disrupted visuospatial perception were generally not able to highlight the presence of neglect symptoms in CRPS patients^10, 17, 29–31^. There are some perceptual difficulties in CRPS that have been reported for the tactile modality, such as decreased tactile acuity of the affected limb^13, 32^, and referred sensations between stimulated body parts^33, 34^, but it is difficult to disentangle the cortical from the peripheral origins of these unilateral tactile perceptual symptoms. For these reasons, the “neglect-like” nature of the cognitive symptomatology in CRPS was called into question. Punt *et al*.^19^, for example, argued that the perceptual difficulties of CRPS patients only emerged from specific laboratory-based measures, suggesting that they might not be clinically relevant. However, some clinical trials successfully alleviated CRPS pain and other related symptoms by using cognitive rehabilitation techniques based on visual perception and visuo-motor coordination manipulation^31, 35–37^. It could thus be counter-argued that cognitive testing instruments classically used to diagnose hemispatial neglect after hemispheric damage are not sensitive enough to clearly characterize spatial perception deficits in CRPS^4, 6^. Furthermore, Moseley *et al*.^38, 39^ succeeded to demonstrate, using a temporal order judgment (TOJ) task, that some tactile perceptual deficits observed in CRPS can obviously be attributed to neuro-cognitive deficits affecting spatial perception. TOJ tasks consist in discriminating the temporal order of two sensory events presented in a rapid temporal succession. Concretely, pairs of stimuli are presented with various delays between them, and participants have to report which of the two stimuli was perceived as presented first. One important parameter of the participants’ performance is the *point of subjective simultaneity* (PSS) and corresponds to the delay between the two stimuli at which the two stimuli have an equal chance to be perceived as being presented first. Significant changes in the PSS can be related to a shift in judgment toward one of the two stimuli, and allow characterizing cognitive biases to the advantage of the perception of one of the two stimuli. Moseley *et al*.^38^ applied pairs of tactile stimuli, one on either hand, and showed that CRPS patients’ perceptual judgments were biased to the detriment of the stimulation on the affected hand. However, this was only observed when the patients’ hands were in a normal, i.e. uncrossed posture. When patients were asked to cross their hands over their body midline, perceptual judgements were biased to the detriment of the unaffected hand, as if the patients’ performance did not rely on the actual stimulation of the affected hand, but rather on the side of space in which the affected hand normally resides. These results clearly show that somatosensory perceptual difficulties in CRPS, as illustrated by TOJ tasks, cannot be attributed to altered peripheral coding or spinal transmission of somatosensory inputs, but rather to deficits in their cognitive processing. In a recent study, Reid *et al*.^17^ replicated these results in the tactile modality, but also showed, with the same TOJ task, that spatial perception of auditory stimuli, i.e. stimuli occurring outside the body space, was not impaired. Auditory stimuli were delivered using speakers placed close to the patients’ hands. The authors concluded that cognitive difficulties in CRPS are limited to the representation of body-related information and the perception of body space, and do not extend to the perception of external space. It can however be argued that the auditory modality is not the ideal modality to test lateralized perceptual difficulties in external space, since audition, conversely to touch and vision, is not primarily spatially coded. Indeed, because both ears are stimulated (especially with external speakers), sound localization is extracted from interaural intensity or time differences^40^. Importantly, presuming that CRPS-related perceptual deficits are limited to the somatosensory modalities would invalidate neuropsychological techniques that use visuospatial attention as a useful method to treat chronic pain.

**Figure 1:**
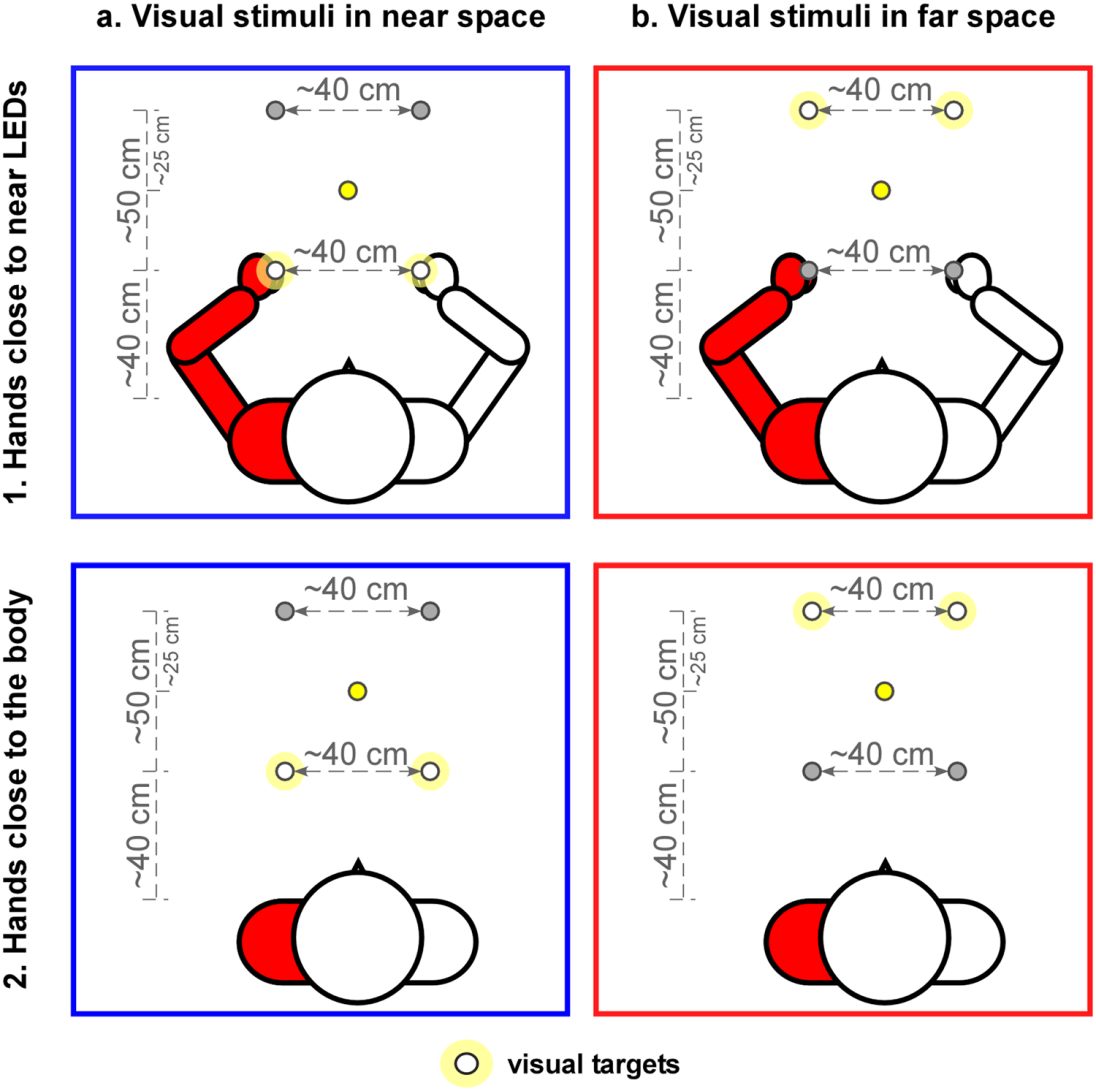
Illustration of the experimental set-up for the visual TOJ task. Visual stimuli are presented by means of two pairs of LEDs, one placed near and the other placed far from the trunk. The participants’ hands are either positioned close to the LEDs in near space (1) or close to the body, on the thighs, next to the trunk (2). In each block, participants performed the task only with one pair of LEDs, either in near (**a**, in blue) or in far (**b**, in red) space. The task-relevant pair is considered as the visual targets and illustrated by the white circles with a slight yellow halo. A centrally-placed yellow LED, represented by the yellow circles, serves as fixation point. The figure depicts a participant affected by left-sided CRPS.

**Figure 2:**
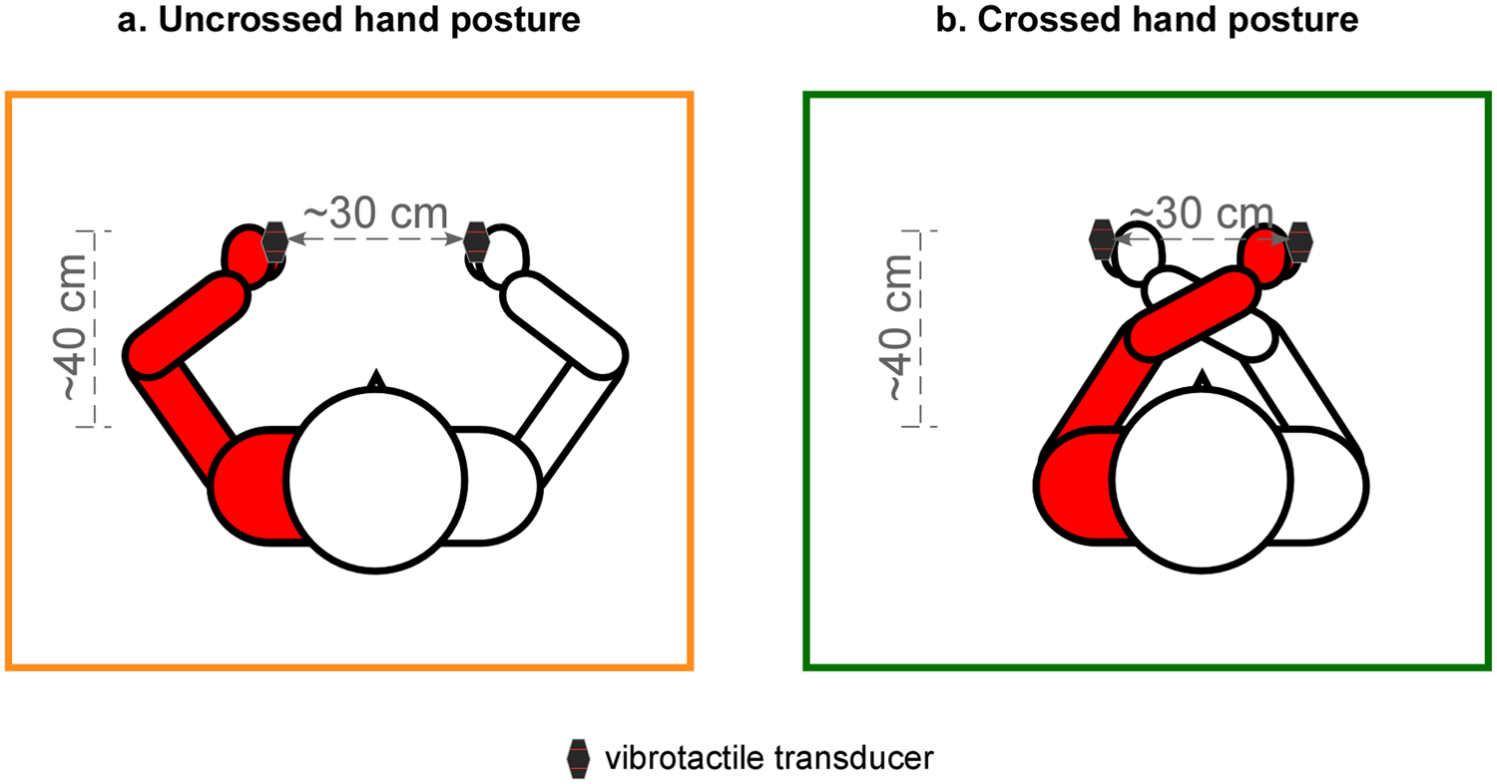
Illustration of the experimental set-up for the tactile TOJ task. Tactile stimuli are generated by two vibrotactile transducers held between the thumb and the index finger of each hand. The hands are either held in an uncrossed posture (**a**) or crossed over the sagittal body midline (**b**). Participants are blindfolded and presented with white noise. The figure depicts a participant affected by left-sided CRPS.

The aim of the present studies was to test whether perceptual deficits in CRPS patients, as reported for somatosensory stimuli^17, 38, 39^, can actually extend to external space, by using TOJ tasks with the presumably more indicated -visual- modality. Sixteen participants affected by upper-limb CRPS participated in the study. In two different series of tasks, they judged the temporal order of pairs of either visual stimuli, presented by light-emitting diodes (LEDs), or tactile stimuli, applied by vibrotactile transducers hold between the thumb and the index fingers of each hand. Although the perception of somatosensory stimuli in CRPS has already been tested in previous studies^17, 38, 39^, we included the tactile TOJ task in the present studies to obtain data for both somatic and non-somatic stimuli in the same sample of patients. In addition, for both the visual and the tactile TOJ tasks, an adaptive method was used (the adaptive PSI method^41^) to vary stimulus levels and to derive the measures of interest. As compared to previous studies which used non-adaptive procedures^17, 38, 39^, the adaptive PSI method has the advantage that the procedure is adapted to each patient’s own performance, which allows to reduce the number of trials while maintaining a precise estimation of the parameters of interest and to minimize floor or ceiling effects (see ref. 42 for a more detailed description). In the visual TOJ tasks, one stimulus was presented in either side of space, i.e. in the side of the affected hand and that of the unaffected hand. Visual stimuli were presented either near or far from the patients’ trunk, with the hands placed either close to the nearest visual stimuli or close to their body (Fig. 1). These manipulations of the locations of the LEDs and the hands were aimed to test the possible dissociation between perceiving the proximal and perceiving the more distal external space (for a review see ref. 43). In the tactile TOJ tasks, one stimulus was applied on either hand, and judgments were performed either with the hands in an uncrossed or a crossed posture (Fig. 2). Changing the posture of the hands was intended to test whether tactile deficits affect an anatomical or a spatial representation of the body^6^. We hypothesized that, for both the visual and the tactile tasks, temporal order would be judged to the advantage of the stimuli presented close to or on the unaffected hand, showing that spatial cognitive deficits observed in CRPS do not only affect the perception of the body, but also extend to the external space surrounding it.

## Results

### Visual TOJ tasks

**Figure 3:**
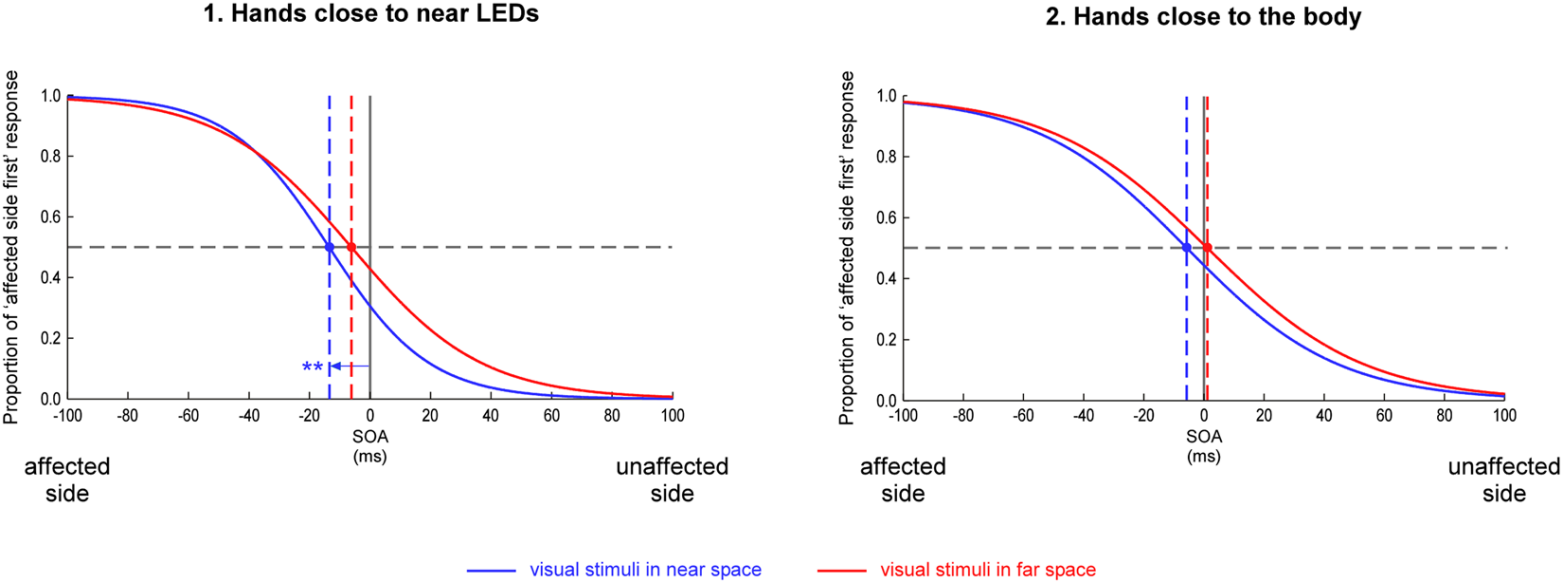
Visual TOJs. The figure illustrates the fitted logistic functions from the data of the 14 participants for the (1) hands close to near LEDs and the (2) hands close to the body position. The x-axis represents different hypothetical SOAs between the two visual stimuli: negative values on the left side indicate that the visual stimulus occurring in the side of space of the affected limb was presented first, while positive values indicate that the visual stimulus occurring in the side of space of the unaffected limb was presented first. The y-axis represents the proportion of trials in which the participants perceived the stimulus presented in the side of space of the affected limb as occurring first. Blue curves represent the conditions in which visual stimuli were presented in near space, with the corresponding PSS values indicated by the blue dashed lines. Red curves represent the conditions in which visual stimuli were presented in far space, with the corresponding PSS values indicated by the red dashed lines. The arrow in (1) indicates the PSS value significantly different from zero (**p ≤ 0.01) for the condition during which visual stimuli were presented in near space with the hands placed next to them.

**Figure 4:**
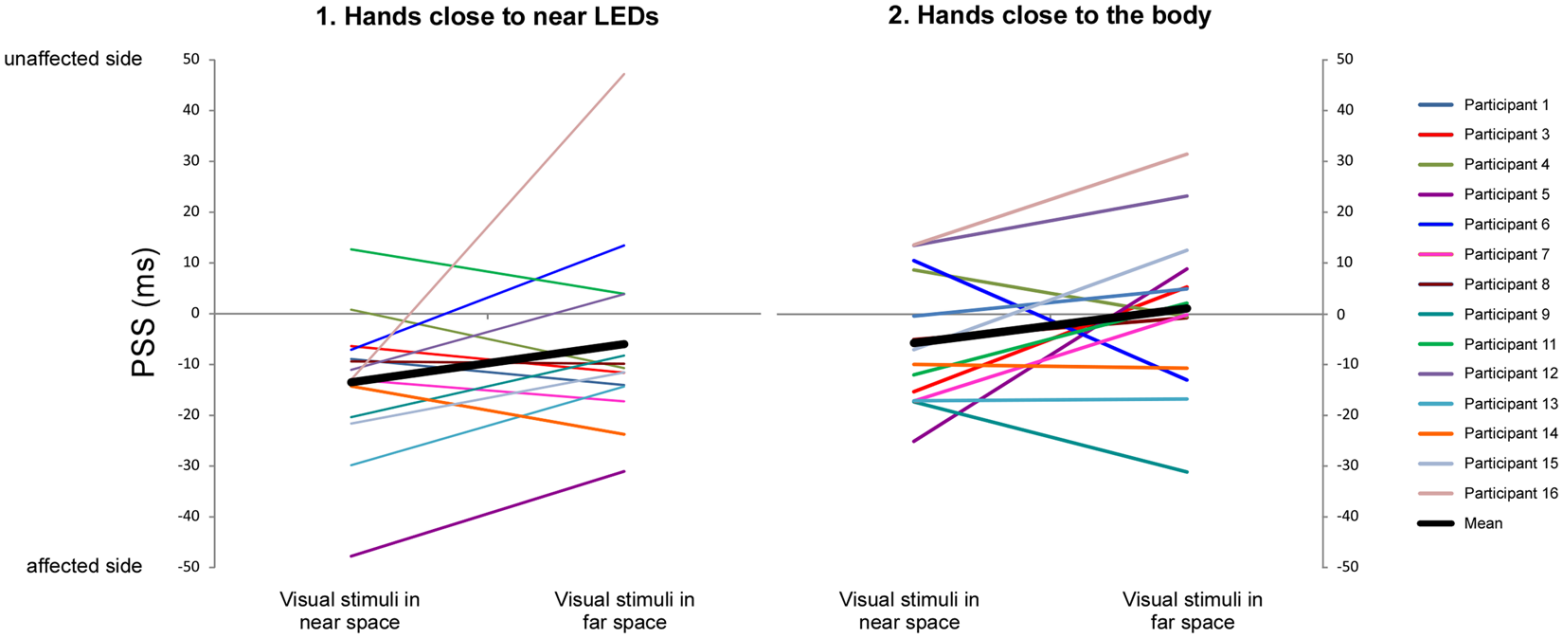
Individual and mean PSS values for the visual TOJ tasks. The left graphic illustrates the PSS values for the conditions with the hands close to the near LEDs (1), and the right graphic the PSS values for the conditions with the hands close to the body (2). Within each graphic, the left side illustrate the PSS values of the tasks performed with the visual stimuli in near space, the right side those with the visual stimuli in far space. Each color line represents one of the 14 participants who participated in the visual TOJ tasks; the thick black lines represent the mean of the PSS values across these 14 participants.

Figure 3 shows the fitted psychometric curves in which the proportion of trials in which the visual stimulus presented in the same side as the affected limb was reported as appearing first was plotted as a function of the time interval between the two visual stimuli (SOA, i.e. stimulus onset asynchrony). The individual and mean PSS scores are illustrated in Fig. 4. Simple t-tests showed that PSS values were only significantly different from zero when visual stimuli were presented in near space and the hands placed next to them (*t*(13) = −3.582, *p* = 0.003, *d* = −0.96), showing that, in this condition, the visual stimulus appearing close to the affected limb had to be presented significantly earlier (M = −13.49, SD = 14.09 ms) than the stimulus appearing close to the unaffected limb to be perceived as occurring simultaneously. This suggests a significant bias at the advantage of the perception of the visual stimulus presented close to the unaffected hand and to the detriment of the visual stimulus presented close to the affected hand. The PSS values of all the other conditions were not significantly different from zero (all *t* ≤ −1.64, p ≥ 0.125). These results were confirmed by Bayesian analyses (see Supplementary materials). When comparing the different conditions, repeated measures ANOVA showed a significant main effect of *hand position* (*F*(1,13) = 5.578, *p* = 0.034, *Ƞ²p* = 0.300): PSS values were larger (i.e. more negative) when the hands were placed close to the LEDs in near space (M = −9.739, SD = 13.854) than when they were placed close to the body (M = −2.236, SD = 12.335). The main effect of *visual stimuli position* approached significance (*F*(1,13) = 4.395, *p* = 0.056, *Ƞ²p* = 0.253), suggesting a trend for larger (i.e. more negative) PSS values for visual stimuli presented in near space with regard to the trunk (M = −9.897, SD = 11.546) than for visual stimuli presented further away (M = −2.401, SD = 14.893). In addition to the results of the t-tests, these findings suggest larger biases to the detriment of the visual stimuli presented in the affected side when the hands are placed close to them. The interaction between these two factors was not significant (*F*(1,13) = 0.012, *p* = 0.914, *Ƞ²p* = 0.001).

As for the slope of the psychometric function, indexing the precision of the participants’ judgments, the repeated measures ANOVA did not reveal significant main effects or interactions (all *F* ≤ 1.032, *p* ≥ 0.328), with an exception for the factor *hand position* which approached significance (*F*(1,13) = 4.518, *p* = 0.053, *Ƞ²p* = 0.258). These results suggest that participants’ judgments were less noisy, i.e. less variable, in the conditions during which the hands were placed close to the LEDs in near space than when they were placed close to the body. This finding support the PSS results reported above, as a steeper slope for the conditions in which the hands are placed on the table, i.e. close to the LEDs in near space, suggests that participant’s judgments were more systematically biased in these specific conditions and thus more precise than in the other conditions.

### Tactile TOJ tasks

**Figure 5:**
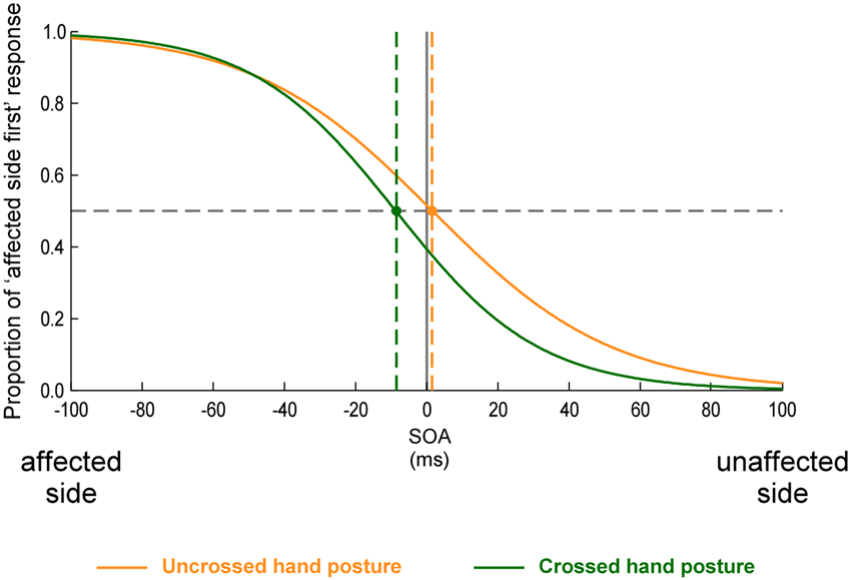
Tactile TOJs. The figure illustrates the fitted logistic functions from the data of the 12 participants. The x-axis represents different hypothetical SOAs between the two tactile stimuli: negative values on the left side indicate that the affected limb was stimulated first, while positive values indicate that the unaffected limb was stimulated first. The y-axis represents the proportion of trials in which the participants perceived the affected limb as being stimulated first. The orange curve represents the conditions in which the hands were held in an uncrossed posture, with the corresponding PSS value indicated by the orange dashed line. The green curve represents the conditions in which the hands were crossed over the sagittal body midline, with the corresponding PSS value indicated by the green dashed line. None of the two PSS values were significantly different from 0.

**Figure 6:**
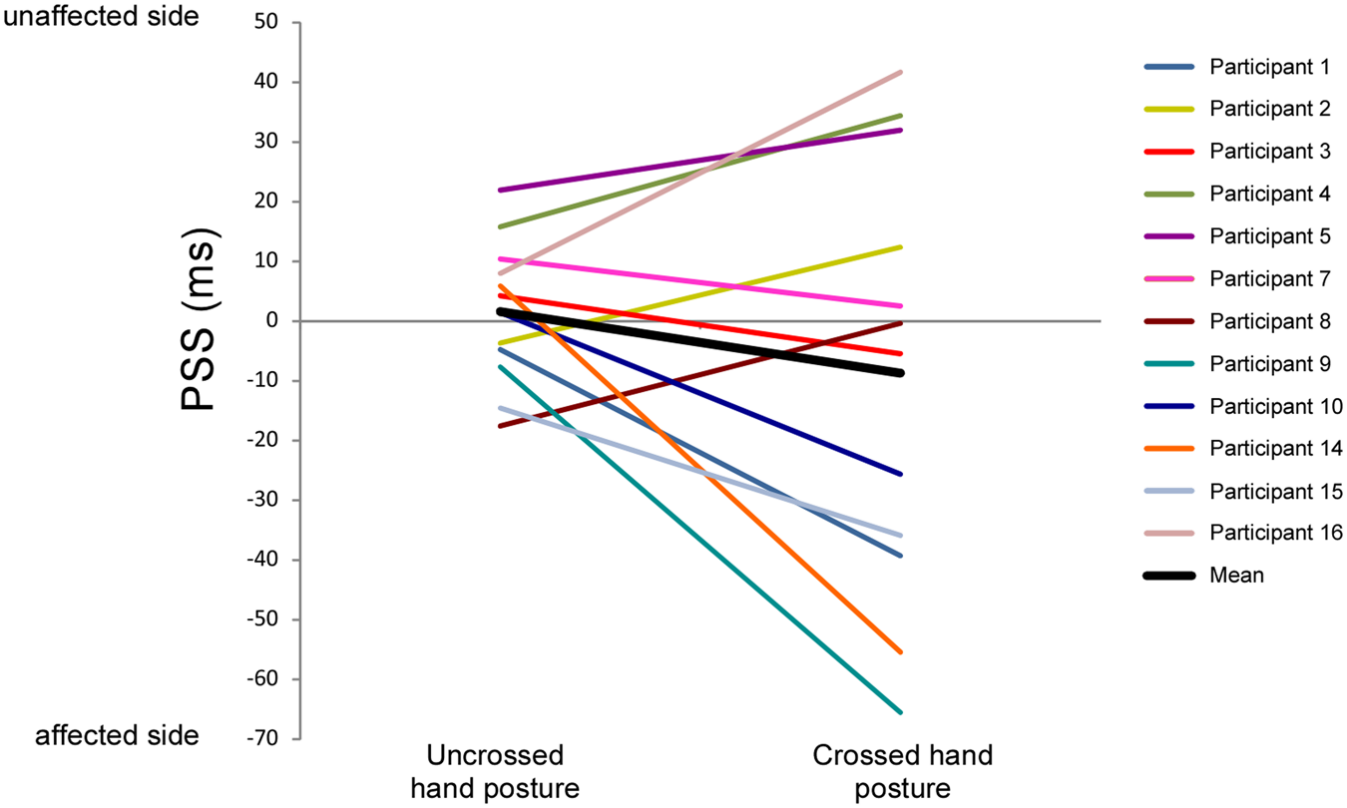
Individual and mean PSS values for the tactile TOJ tasks. Within the graphic, the left side illustrates the PSS values for the tasks performed with the hands in an uncrossed posture, the right side those with the hands in a crossed posture. Each color line represents one of the 12 participants who participated in the tactile TOJ tasks; the thick black line represents the mean of the PSS values across these 12 participants.

Figure 5 shows the fitted psychometric curves in which the proportion of trials in which the tactile stimulus applied on the affected limb was reported as appearing first was plotted as a function of SOA. The individual and mean PSS scores are illustrated in Fig. 6. None of the PSS values were significantly different from 0 (all *t*(11)  ≤ −0.841, p ≥ 0.418). These results were confirmed by Bayesian analyses (see Supplementary materials). There was no significant difference between the uncrossed and the crossed hand posture as shown by the repeated measures ANOVA (*F*(1,11) = 1.342, *p* = 0.271, *Ƞ²p* = 0.109).

Regarding the slope values of the psychometric function, this difference was also not significant (*F*(1,11) = 0.351, *p* = 0.565, *Ƞ²p* = 0.031).

## Discussion

In the present studies, we investigated whether CRPS affects spatial perception, i.e. induces attentional biases to one side of space, by means of TOJ tasks. Attentional effects on TOJ have been explained by the law of prior entry, which postulates that attended stimuli are perceived earlier than unattended ones (for a review see ref. 44). When stimuli are presented in opposite sides of space, prior-entry related spatial biases in TOJ are typically evidenced by shifts in PSS values. These shifts indicate that, in order to have the chance to be perceived as stimulated first equally often, one side of space has to be stimulated before the opposite side, resulting from a facilitated perception of the stimuli presented in the latter one. In the present experiments we used two series of TOJ tasks, one with tactile stimuli applied to CRPS patients’ hands and another one with visual stimuli presented at various distances from the patients’ hands. The aim was to clarify whether CRPS patients’ perceptual biases are limited to stimuli applied to the patients’ body, as previously shown^17^, or if they also extend to stimuli occurring in external space. In contrast to previous studies^17, 38, 39^ we did not evidence any systematic changes in the PSS values in tactile TOJs, neither in the uncrossed nor in the crossed hand posture. We did however succeed to demonstrate significant changes in visual TOJs. More specifically, we showed that visual stimuli directly surrounding the affected limb were perceived significantly later than stimuli that were presented next to the unaffected hand, suggesting that participants paid less attention to visual stimuli occurring close to the affected hand. Furthermore, PSS shifts were in general more important when the hands were placed on the table (i.e. close to the nearest visual stimuli) than when they were placed close to the body. The difference between the near and far position of the visual stimuli approached significance. Taken all together, the results of the visual TOJs indicate that the perception of external stimuli is affected, especially when they occur in the space immediately surrounding the CRPS patients’ hands. These deficits affect the perception of visual stimuli occurring close to the affected limb, as if the patients positively biased the perception of the visual stimuli occurring in the opposite side of space.

Spatial perception is a complex cognitive ability as it encompasses different cognitive mechanisms with distinct underlying neural processes (for a review see ref. 43). These cognitive mechanisms are based on specific frames of reference that are defined as coordinate systems to map the position of a stimulus in a particular portion of space. For instance, the position of a somatosensory stimulus on the cutaneous space can be located based on anatomical projection of the receptors fields to spatially organized subgroups of neurons in somatosensory cortical areas (somatotopic reference frame), but also based on the relative position of each limb in external space (spatiotopic reference frame). The latter one allows recognizing that, when the hands are for instance crossed and the eyes closed, a touch applied on the left hand results from the contact of an object coming from the right part of space (e.g. refs 45, 46). Similarly, the ability to perceive visual stimuli in external space is also regulated by dissociated reference frames, with the peripersonal reference frame for external stimuli occurring in the proximity of the body, and the extrapersonal reference frame for stimuli occurring farther away (e.g. refs 47, 48). The peripersonal reference frame is of primary importance as it allows integrating body and extra-body information to form a coherent representation of the body space and its immediate surrounding space. This multimodal representation of the body and the space nearby facilitates the interaction with objects in contact with the body, such as the manipulation of non-threatening objects^49^ and defensive reactions against threatening objects^6^. Indeed, to adequately react to a potential damaging stimulus, it is necessary to locate the part of the body that is potentially being harmed, the position of the threat in external space, as well as the relative postural position of the threatened limb in space. It has been shown, for example, that the perceived pain induced by a nociceptive stimulus can depend on body posture and the vision of the limb on which the nociceptive stimulus is applied^50–52^ (however, see refs 53, 54). For these reasons the existence of a spatial coding for nociceptive stimuli according to a peripersonal reference frame has been suggested and highlighted in recent studies^55–57^. For instance, De Paepe *et al*.^56^ have shown that the temporal perception of nociceptive stimuli applied on the hands of healthy volunteers is affected by the occurrence of visual stimuli, especially when they are presented close to the stimulated hands. Similarly, we recently demonstrated, still with healthy volunteers, that TOJs of visual stimuli can be biased to the advantage of the visual stimulus presented close to the hand on which a nociceptive stimulus is applied^42^. These results support the idea that visual experience of the immediate surrounding of the body influences how we perceive bodily sensations and represent our body, as well as that the way we perceive our environment can also be shaped by bodily information like pain. The present studies indicate that the development of a chronic pain condition such as CRPS can also have an impact on how patients perceive their environment (see refs 58–60 for similar conclusions in limb immobilization and amputation).

Our results are at odds with those observed in previous studies. Reid *et al*.^17^ showed that CRPS affects TOJs of tactile stimuli applied on the hands, but not those of auditory stimuli presented close to the hands. They concluded that spatial cognitive deficits were limited to bodily-specific sensations and do not affect the perception of external space. However, as outlined in the introduction, tones delivered by means of external speakers are not ideal to explore subtle spatial biases, as both ears are stimulated by all tones, independently of the original speaker. Measuring saccade latencies in a visuospatial attentional cueing task, Filippopulos *et al*.^61^ come to the same conclusion as Reid *et al*.^17^, since they didn’t observe any differences between CRPS patients’ and controls’ performances. However, in the study of Filippopulos *et al*.^61^, visual stimuli were presented on a screen placed at a distance of 68.5 cm from the participant’s body. This distance is even greater than the distance of the visual stimuli presented in far space in our studies and for which attentional biases were not significantly different from 0. Since, in our studies, spatial attention was only significantly shifted towards the unaffected side when visual stimuli were presented in the immediate vicinity of the limbs, it seems conceivable to postulate that spatial perception of external stimuli is distorted in CRPS if the stimuli are processed according to frames of reference that include interactions with the body.

The most striking difference relative to previous experiments is that we did not succeed to replicate the spatial biases in the tactile modality observed with similar TOJ tasks^17, 38, 39^. However, the lack of significant results doesn’t allow us to conclude that there is no somatosensory perceptual bias in CRPS patients. Instead, we were not able to evidence a systematic bias toward one of the two sides of space at the group level, as some of our patients displayed biases in opposite directions, which could furthermore change from one condition to the other in a same patient. One reason for these discrepant results could be that, compared to the other studies, the participants of our study seem on average less chronic, a fact that could imply differences at the level of cortical reorganization. However, this does not appear to be the main reason since even less chronic patients showed some signs of cortical reorganization, as demonstrated by the presence of significant visuospatial biases. Symptoms in CRPS are thought to “centralize” gradually, with a time frame that varies from patient to patient^62^. Furthermore, duration of CRPS did not significantly predict the results of our TOJ tasks (see Supplementary materials). Since other clinical measures, such as differences in hand volume or temperature of the hands, average pain over the two last days, disability score of the Disabilities of the Arm, Shoulder and Hand (DASH) questionnaire and degree of immobilization, were not related to the measure of tactile bias (see Supplementary materials), these measures seem also not to explain the discrepancies with the literature. Some of the patients we tested also presented with pain in other parts of the body (see Table 1), which could have influenced their performance in the different tasks. However, all of them described these sensations as rather minor and much less constraining as compared to the pain in the CRPS limb. Furthermore, these participants didn’t seem to perform differently from the others.

**Table 1:** *Participant characteristics*

ID	Age/sex/ handedness	Inciting injury	CRPS limb	Diagnosis	Dur	Current treatment/medication	Other pain	Other	Task
1	37/F/R	STI wrist	L	*NS*	15	PT	/	/	V,T
2	47/F/R	STI wrist	L	CRPS-R	32	PT/OT/Amitriptyline	/	Glaucoma	T
3	44/M/R	Frac thumb	L	*NS*	5	PT	/	/	V,T
4	46/M/R	Frac-PS wrist	R	*NS*	3	PT	/	/	V,T
5	65/F/R	Frac-PS wrist	L	CRPS-C	6	PT/Paracetamol,Ibuprofen	/	/	V,T
6	52/F/R	STI cut nerve-PS	L	CRPS-C II	3	PT/Paracetamol,Pregabalin	/	/	V
7	49/M/R	Frac fingers	R	CRPS-C	2.5	PT	/	/	V,T
8	38/F/R	STI wrist	R	CRPS-C	6	PT	L hand, R ankle	/	V,T
9	63/F/R	Frac-PS wrist	R	CRPS-C	7	Ibuprofen	/	/	V,T
10	56/F/ambi	Frac shoulder	R	CRPS-R	3	PT/Amitriptyline,Tramadol	L thumb	Amblyopia	T
11	54/F/L	Frac wrist	L	CRPS-R	4	PT	R shoulder	/	V
12	38/M/R	Frac wrist	R	CRPS-R	9	PT	/	/	V
13	47/F/R	PS wrist	R	CRPS-C	18.5	PT/OT/Pregabalin,Topiramate,Duloxetine	/	/	V
14	48/F/R	STI hand	L	CRPS-R	17	PT/OT/Amitriptyline	/	/	V,T
15	61/F/R	Frac-PS wrist	L	CRPS-R	6	PT/OT/Tramadol	L foot	/	V,T
16	54/F/R	PS hand	R	CRPS-C	10	PT/OT/Tramadol	/	/	V,T

Note. Age in years; F = female; M = male; R = right; ambi = ambidextrous, L = left; STI = soft tissue injury; Frac = fracture, PS = post-surgery; *NS* = not specified; CRPS-R = Budapest research criteria for CRPS; CRPS-C = Budapest clinical criteria for CRPS; II = CRPS due to nerve injury; Dur. = duration since inciting injury in months; PT = physical therapy; OT = occupational therapy; V = participated in visual task; T = participated in tactile task.

The observed discrepancies, not only between TOJ studies, but also in the literature about CRPS-related cognitive deficits in general, raise the question of important inter-individual differences in the cognitive symptomatology of CRPS. Looking carefully at the literature on post-stroke hemispatial neglect, it appears that the symptomatology is highly variable. Neglect can affect different sensory modalities separately or concurrently and visuospatial deficits can impact or spare motor exploration^20, 27^. Dissociations have also been observed between the different frames of reference (for a summary see refs 20, 63), as well as between perceptual and representational abilities^23^. The variability of the symptoms may be accounted for by the variability of the location and the extent of the lesions. Similar dissociations can also be observed in CRPS patients, as Sumitani *et al*.^36, 64^ demonstrated biases in visuospatial attention *in favor* of the affected limb when patients judged the position of a visual stimulus according to their own body midline (see however refs 30, 31 for different results). We would thus like to propose that the observed discrepancies across studies might be the result of an extreme variability of cognitive difficulties in CRPS, with possible dissociations between different cognitive abilities. Indeed, it has been suggested that cognitive deficits in CRPS are the result of a maladaptive cortical reorganization induced by (implicit) behavioral strategies developed by the patients to avoid the provocation of pain. It seems moreover reasonable to suppose that various strategies can be developed across CRPS patients, which, in turn, might impact cortical plasticity differently. This emphasizes the need to move to a more individual approach rather than a global one when investigating cognitive reorganization in CRPS in the future.

A limitation of the present study is the small size of the sample of CRPS patients we tested. Although previous studies that used TOJ tasks to measure perceptual difficulties in CRPS tested a similar number of patients as in our study^17, 38, 39^, future studies should try to replicate the present and previous findings with a more important sample size. A larger sample size could be possibly of interest to test whether the effects (such as the distance from the visual stimuli from the patients’ trunk) that approached significance in the present study could be considered as significantly modulating the perception of visual stimuli in CRPS. Future studies should also investigate whether the demonstrated effects of the position of the patients’ hands on TOJ relates to the fact that in one hand position patients can see their hands whereas in the other hand position they don’t. Furthermore, we have to specify that not all of the patients were diagnosed according to the same criteria and that the sample of patients of the present study could therefore be considered as being heterogeneous. Of the 13 patients that were diagnosed with the Budapest clinical criteria for CRPS, 6 also fulfilled the Budapest criteria for research^65^. Three other patients were diagnosed according to non-specified criteria (see Methods section). One has however to note that only one more additional symptom with regard to the clinical criteria is required to fulfil the research criteria. Furthermore, in the validation study of Harden *et al*.^65^ it has also been shown that the advantages of the research criteria over the clinical criteria are minimal. A sample of patients that fulfil either clinical or research Budapest criteria seems thus after all not that heterogeneous. As to the 3 patients that were not diagnosed according to the Budapest criteria, we still decided to keep them in the sample as they were diagnosed by experienced orthopaedic surgeons after several careful clinical examinations which were corroborated by bone scintigraphy. Testing a sample of patients that may seem in some ways heterogeneous can be considered as reflecting the everyday clinical diversity that many clinicians are confronted with. In our opinion, testing patients with inclusion/exclusion criteria that are too strict bears the risk to only test a very specific, very homogenous sample of patients, which could difficultly be considered as representing clinical reality.

To conclude, the present experiments demonstrated the presence of visuospatial deficits in CRPS patients. Conversely to previous statements (e.g. ref. 19), our results highlight the need to develop more specific empirical designs to uncover cognitive difficulties in CRPS patients. Similar conclusions have been proposed for hemispatial neglect, since it has been observed that some post-stroke patients still display difficulties in their daily life while standard tests of neglect don’t reveal any symptoms (e.g. ref. 66). This is particularly important for the development and individual orientation of rehabilitation strategies which aim at integrating physiotherapeutic and neuropsychological aspects to treat chronic pain.

## Methods

### Participants

Sixteen participants affected by upper-limb CRPS took part in this study (Table 1). Sample size was determined based on previous studies that used TOJ tasks with CRPS patients^17, 38, 39^. Exclusion criteria were any neurological and severe psychiatric disorders and unresolved orthopedic injuries. Uncorrected vision difficulties were considered as exclusion criteria for the visual TOJ task. For the tactile task only, nerve damage (CRPS type II) or any medical condition disrupting somatosensory perception were also considered as exclusion criteria. Thirteen participants fulfilled the Budapest clinical criteria for CRPS, of whom six also fulfilled the Budapest criteria for research^65^. Three participants were diagnosed according to non-specified criteria by orthopaedic surgeons based on repeated clinical examination and corroborated by bone scintigraphy^62^. For the visual task, two participants were excluded because of visual impairments (one case of glaucoma and one of amblyopia). The mean age of the remaining 14 participants (10 women) was 49 years (SD = 9, range: 37–65 years). Half of them presented with a left-sided CRPS. All but one of the participants were right-handed according to the Flinders Handedness Survey (Flanders)^67^. For the tactile task, four participants were excluded from statistical analysis, one participant because of nerve damage associated with the CRPS and three participants based on their poor performance (see Data analysis section for details). The remaining 12 participants (nine women) had a mean age of 50 years (SD = 9, range: 37–65 years), six of them presenting with a CRPS of the left side. Eleven participants were right-handed and one was ambidextrous according to the Flanders questionnaire. The experimental procedure was approved by the local ethic committee (Commission d’Ethique Biomédicale Hospitalo-Facultaire de l’Université catholique de Louvain) in agreement with the latest version of the Declaration of Helsinki and was carried out in accordance with the corresponding guidelines and regulations. All participants signed an informed consent form prior to the experimental session. Participants received financial compensation for their participation.

### Stimuli and apparatus

Visual stimuli were presented by means of four white light emitting diodes (LEDs) with a 17-lm luminous flux, a 6.40-cd luminous intensity, and a 120° visual angle (GM5BW97330A, Sharp Corporation, Japan). They were illuminated for 5 ms and perceived as brief flashes. Participants were familiarized with the stimuli by reporting the position of the flashing LED (i.e. the left or the right one, close to the body or farther away). An additional yellow LED (min. 0.7 cd luminous intensity at 20 mA, 120° viewing angle) was used as fixation point during the visual task (Multicomp, Farnell element14, UK).

The LEDs were fixated on the surface of a table. Two of the white LEDs were placed ∼40 cm away from the participant’s trunk and with a distance of ∼40 cm between them (visual stimuli in near space). The other two LEDs were positioned ∼50 cm from the LEDs in near space, with a distance of ∼40 cm between them (visual stimuli in far space). The yellow fixation LED was placed equidistantly from the four white LEDs at a distance of ∼65 cm in front of the participants’ trunk (Fig. 1).

Vibrotactile stimuli were generated by two vibrotactile transducers driven by standard audio amplifiers (TL-002–14R Haptuator Redesign, Tactile Labs, Inc., Montreal, Canada). Stimulus vibration were lasting 10 ms, at 440 Hz. Participants were familiarized with the stimulations and, if necessary, signal amplitude was adapted individually, in order to match the perceived sensation and intensity between the left and right hand stimuli. During the task, participants held a vibrotactile transducer between the thumb and the index fingers of each hand. The two transducers were held with a distance of ∼30 cm between them, at a distance of ∼40 cm from the trunk and without touching the table (Fig. 2).

### Procedure

General assessments and measures that were taken before the experimental session, as well as between the different experimental blocks, are reported in Supplementary materials. The two TOJ tasks were always administered during the same day (with a break of at least 30 min between them) and the order of presentation of the two tasks was counterbalanced across participants.

#### Visual TOJ task

Participants were sitting in a dimly-illuminated testing room, in front of the table with the LEDs. In half of the blocks their hands were placed on the table with palms down next to the LEDs in near space (i.e. the left hand placed next to the left LED and the right hand next to the right LED). Each of these two LEDs was positioned at a maximum distance of 1 cm from the metacarpophalangeal joint of the index finger. In the other half of the blocks, the participants held their hands in a comfortable position close to their body, on their thighs, next to the trunk (Fig. 1). Their heads were stabilized with a chin-rest placed approximately 10 cm from the trunk, in order to minimize head movement.

A trial started with the illumination of the fixation point. After 500 ms a pair of visual stimuli appeared, one stimulus presented in either side of space. Depending on the block, the pair of stimuli was presented either in near or in far space. Twenty possible time intervals (SOAs for stimulus onset asynchronies) were used between the two visual stimuli of the pair: ± 200, ± 145, ± 90, ± 75, ± 60, ± 45, ± 30, ± 15, ± 10, ± 5ms (negative values indicate that the left LED was illuminated first). Participants were instructed to keep their gaze at the fixation point during the whole trial. In half of the blocks participants had to respond verbally which stimulus they perceived as occurring first, whereas they had to respond which stimulus was perceived as occurring second in the other half of the blocks (by answering ‘*left*’ or ‘*right*’). These two response modalities were used to minimize response biases that can be confounded with genuine perceptual biases (for a discussion see refs 44, 68). No specific instruction was given regarding response speed. Illumination of the fixation point was switched off as soon as the participant’s response was encoded by the experimenter. The next trial started 2000 ms later. No feedback regarding the accuracy of the participant’s performance was given.

Participants started with a practice session of four blocks of 10 trials each, one block per condition (i.e., LEDs in near vs. far space, and *‘which is first*’ vs. ‘*which is second*’ responses), but only with the two highest SOAs. The experimental session consisted of eight blocks, resulting from the combination of the factors *hand position* (close to near LEDs vs. close to the body), *visual stimuli position* (near space vs. far space) and *response modality* (‘*which is first*’ vs. ‘*which is second*’). The order of the blocks was counterbalanced for the two hand positions (half of the participants started the experiment with all the blocks *hands close to near LEDs*, the other half with all the blocks *hands close to the body*), and randomized for the visual stimuli positions and response modalities. Each block was composed of 40 trials and for each trial the presented SOA was determined online according to the adaptive PSI procedure^41^, i.e. based on participants’ performance on all previous trials (implemented trough the Palamedes Toolbox^69^). With this procedure the parameters of interest (see Measures section) are estimated validly and reliably without probing extensively all the possible SOAs, and presented SOAs are adapted to each participant’s own performance (see also ref. 42 for a detailed discussion of the advantages of using the adaptive PSI method in TOJ experiments). A rest period between the blocks was possible when requested. Duration of the visual TOJ experiment was ∼60 min.

#### Tactile TOJ task

Participants were sitting in front of the same table and held a vibrotactile transducer between the thumb and the index finger of each hand. In half of the blocks their hands were in an uncrossed posture, while in the other half of the blocks they held their hands crossed over the sagittal body midline. For the crossed posture, all participants placed their affected arm over the unaffected arm, since the reverse was too painful for the majority of participants (Fig. 2). Participants’ heads were placed in a chin-rest as for the visual TOJ experiment. To mask any sound produced by the vibrotactile stimulators, white noise was presented continuously through headphones. Participants were also blindfolded throughout the different blocks to avoid any visual distraction.

On each trial two vibrotactile stimuli were presented, one on each hand. Twenty possible SOAs were used between the two stimuli: ± 200, ± 145, ± 90, ± 75, ± 60, ± 45, ± 30, ± 15, ± 10, ± 5ms (negative values indicate that the left hand was stimulated first). Participants had to respond verbally which hand they perceived as being stimulated first in half of the blocks, whereas they had to respond which hand was perceived as being stimulated second in the other half of the blocks (by answering ‘*left*’ or ‘*right*’). No specific instruction was given regarding response speed. The response was encoded by the experimenter and the next trial started 2000 ms later. No feedback regarding the accuracy of participants’ performance was given.

Participants started with a practice session of 4 blocks of 10 trials each, one block per condition (i.e., uncrossed vs. crossed and ‘*which is first*’ vs. ‘*which is second*’) only with the two highest SOAs. The experimental session consisted of 4 blocks, of 40 trials each, resulting from the combination of the factors *hand posture* (uncrossed vs. crossed) and *response modality* (‘which is first’ vs. ‘which is second’). The order of the blocks was counterbalanced for the two hand postures (half of the participants started with the uncrossed hand posture, whereas the other half started with the crossed hand posture) and randomized for the response modalities. As for the visual TOJ, presented SOAs were determined according to the adaptive PSI procedure^41^. A rest period between the blocks was possible when requested. Duration of the tactile TOJ task was ∼30 min.

### Measures

Data were fitted with a logistic function for each participant and for each condition in order to compute two parameters of interest, α and β. The α defines the overall position of the curve along the abscissa^70^, i.e. the threshold of the function. In our study, the threshold corresponds to the SOA at which the proportion of trials in which the visual stimulus presented at the same side as the affected limb (or the tactile stimulus applied on the affected hand) was reported as presented first, reaches the 0.5 criterion. Stimuli that were initially presented on the left during the tasks were recoded as corresponding to stimuli presented in the affected side of space/on the affected limb. Accordingly, data from participants affected by right-sided CRPS were transformed (multiplied by −1) to facilitate data display and statistical analyses. The α corresponds to the point of subjective simultaneity (PSS) reported in typical TOJ experiments, defined as the amount of time one stimulus has to precede or follow the other in order for the two stimuli to be perceived by the participant as occurring simultaneously^44^. The β parameter defines the slope of the logistic function, which describes the noisiness of the results and can be related to the precision of participants’ responses during the experiment^70^. In most TOJ experiments, the β, i.e. slope of the psychometric function, is used to derive the just noticeable difference (JND). The JND denotes the delay/SOA between two stimuli needed for participants to perceive the correct order of presentation of the two stimuli in a certain percentage of trials^44^. In order to estimate the logistic function we used the adaptive PSI method^41^, which adopts a Bayesian framework, with the ultimate goal of estimating the posterior probability of the parameters of interest without probing extensively all the SOAs. Since a Bayesian approach is used, a prior probability distribution also needs to be postulated, i.e. the researcher’s knowledge/beliefs regarding the values of the parameters of interest^70^. Based on previously conducted pilot studies and existing literature (e.g. refs 38, 39) we used a prior distributions of 0 ± 20 and 0.06 ± 0.6 for the threshold and slope parameters, respectively.

For each condition of the visual TOJ task, the proportion of trials in which the visual stimulus presented at the same side as the affected limb was reported as appearing first was plotted as a function of SOA. For the tactile TOJ, the proportion of trials during which the tactile stimulus applied on the affected hand was reported as being presented first was plotted as a function of SOA for each condition.

### Data analysis

Participants’ data were excluded from statistical analyses if the slope of the psychometric function couldn’t be reliably estimated during the 40 trials, indexing that their performance was below chance level. This criterion led to the exclusion of 3 participants for the analyses of the tactile TOJ.

Before statistical analyses, data from the two response modalities (‘*which is first*’ & ‘*which is second*’) were merged to reduce potential response biases. Firstly, simple t-tests were performed to compare the different PSS values to 0, in order to test the presence of a significant bias related to the affected limb. Evidence for the null hypothesis was quantified by using Bayesian analyses and is reported in the Supplementary materials. Next, the PSS and slope values from the different conditions were compared using an ANOVA for repeated measures. Regarding the visual TOJ, the ANOVA was performed with *visual stimuli position* (near space vs. far space), *hand position* (close to near LEDs vs. close to the body) as within-participant factors. As for the tactile TOJ, *hand posture* (uncrossed vs. crossed) was defined as within-participant factor. These ANOVAs were initially performed with a group factor characterizing which hand was affected (either left vs. right or dominant vs. non-dominant). Since the group factors did not differently impact the results, these analyses are reported in the Supplementary materials.

Greenhouse-Geisser corrections of degrees of freedom and contrast analyses were used when necessary. Effect sizes were measured using Cohen’s d or partial Eta squared. Significance level was set at p ≤ 0.05.

### Data availability

The datasets generated during and/or analysed during the current study are available from the corresponding author on reasonable request.

## Electronic supplementary material

Supplementary materials
