# Supplementary material for: Biased visuospatial perception in complex regional pain syndrome
Source: Sci Rep. 2017 Aug 29;7:9712. doi: 10.1038/s41598-017-10077-8 (PMC5574889; doi:10.1038/s41598-017-10077-8)
Supplement: Supplementary file 1 — Supplementary materials [file 41598_2017_10077_MOESM1_ESM.pdf]

Biased visuospatial perception in complex regional pain syndrome

Lieve Filbrich, Andrea Alamia, Charlotte Verfaille, Anne Berquin, Olivier Barbier, Xavier Libouton, Virginie Fraselle, Dominique Mouraux and Valéry Legrain

Supplementary materials

1. Additional assessments and measures

Before and during the experiments, other parameters were measured with the aim to investigate potential relationships between CRPS symptoms/characteristics and cognitive deficits.

Methods. Participants were assessed with a clinical interview and the Budapest criteria were (re)verified (with the exception of the 5 first participants we tested) (supplementary table S1). Before the experimental session, several additional clinical measures were taken, with the aim to relate them to the cognitive data during statistical analyses (supplementary table S2). The volume of the two hands was used as an index of swelling, measured by means of a hand volumeter (FEI 12-3504 Baseline© Volumetric Measuring Device, Forearm Set, 6X6X24 Inch Cavity). Participants were instructed to put their hand up to the crease of the wrist for 15 sec into the water. The mean of three consecutive measures of displaced water was calculated and converted in ml for both the affected and the unaffected hand[1]. Skin temperature of both the affected and unaffected hand was measured from the hands' dorsa (at the location of the first dorsal interosseous muscle) with an infrared thermometer (Tempett, SENSELab, Sweden). The absolute difference between the affected and the unaffected hand for hand volume as well as for skin temperature was used for statistical regression analyses. Participants also completed the Disabilities of the Arm, Shoulder and Hand (DASH) questionnaire (Institute for Work & Health, Canada) to assess physical symptoms and participants' abilities to

perform daily-life activities. The final score calculated from the 30 items was used for statistical analyses. Average pain over the last 2 days was rated on a visually presented numeric rating scale (NRS) (ranging from 0-10, with 0= no pain and 10= worst pain imaginable). Degree of immobilization and physical activity was assessed with accelerometers (ActiGraph ASP-BTLE 2GB Activity Monitor, ActiGraph, United States) for 7 of the participants. Only participants who returned to the laboratory for other measures and tasks (not reported in this paper) wore the accelerometers for 3 consecutive days. Average vector magnitude count for the affected side minus that for the unaffected side was calculated.

Furthermore, for both the visual and the tactile task, subjective pain intensity and skin temperature of both hands were assessed before and after each block. Pain intensity was assessed by means of a visually presented NRS (ranging from 0-10, with 0= no pain and 10= worst pain imaginable). Skin temperature was measured from the hands' dorsa by means of an infrared thermometer (Tempett, SENSELab, Sweden).

Analyses. Linear regression was used to test if PSS values in the different conditions of the visual and the tactile TOJ tasks could be predicted by the additional clinical measures, i.e. differences in hand volume or temperature of the hands, average pain over the two last days, disability score of the DASH, degree of immobilization and CRPS duration. Differences in hand temperature before and after the different conditions for each hand were tested using a repeated-measures ANOVA, with *hand* (unaffected vs. affected), *time* (before vs. after), *visual stimuli position* (near space vs. far space) as well as *hand position* (close to near LEDs vs. close to the body) as within-participant factors and *side of symptoms* (left vs. right) as between-participants factor for the visual TOJ task. *Hand* (unaffected vs. affected), *time* (before vs. after) as well as *hand posture* (uncrossed vs. crossed) were used as within-participant factors and *side of symptoms* (left vs. right) as between-participant factor for the tactile TOJ task. The differences in pain intensity ratings before vs. after the experimental

blocks were compared between the different conditions using the non-parametric Friedman test.

Results. For the visual TOJ tasks, PSS values for the condition in which visual stimuli were presented in near space and the hands placed next to them could not be predicted by any of the additional clinical measures (all $r^2 \leq .095$, $F \leq 1.264$, $p \geq .283$), with an exception for the measure "difference in hand volume" which approached significance ($r^2= .260$, $F(1,12)= 4.223$, $p= .062$). In the condition in which visual stimuli were presented in far space and the hands were placed next to the near LEDs, PSS values could be related to the measure "difference in hand temperature" ($r^2= .362$, $F(1,12)= 6.810$, $p= .023$), but to none of the other clinical measures (all $r^2 \leq .324$, $F \leq 2.392$, $p \geq .176$). PSS values in the conditions in which the hands were placed close to the body were not related to any of the clinical measures (all $r^2 \leq .466$, $F \leq 2.392$, $p \geq .093$), with an exception for the measure "difference in hand temperature" which approached significance when the hands were placed close to the body and visual stimuli were presented in near space ($r^2= .274$, $F(1,12)= 3.328$, $p= .055$). For the tactile TOJ tasks, none of the additional clinical measures could predict the PSS values, neither in the uncrossed nor the crossed hand posture (all $r^2 \leq .442$, $F \leq 2.879$, $p \geq .121$).

Regarding hand temperatures during the visual TOJ task, the ANOVA revealed a significant main effect of *time* ($F(1,12)= 19.042$, $p= .001$, $\eta^2 p= .613$) and a significant *time x hand position* interaction ($F(1,12)= 18.151$, $p= .001$, $\eta^2 p= .602$). Contrast analyses showed that the two hands were in general warmer after the blocks than before, but only when the hands were placed close to the body (all $t \geq -1.628$, $p \leq .021$). None of the other main effects or interactions reached significance (all $F \leq 3.943$, $p \geq .070$). The difference in pain intensity ratings between the different conditions was not significant ($\chi^2(3)= 2.444$, $p= .485$). For the tactile TOJ tasks, the ANOVA performed on hand temperatures did not show any significant

main effects or interactions (all $F \leq 2.409$, $p \geq .152$). There were also no significant differences in pain intensity ratings between the two hand postures ($\chi^2(1)= 1.286$, p= .257).

2. Additional analyses of the TOJ tasks

2.1 Quantifying the evidence for the null hypotheses

Analyses. To corroborate our analyses with simple t-tests to test the presence of significant biases, we also performed a Bayesian analysis (Bayesian One Sample t-test – JASP Team (2017). JASP (Version 0.8.1.1)) on the PSS values of the four conditions in the visual TOJ task and for the uncrossed and the crossed hand position in the tactile TOJ task. We computed a Bayes factor (BF_{10}, Cauchy prior width: 0.707) to quantify the evidence for the null hypothesis (bias absent) as compared to the alternative hypothesis (bias present)[2].

Results. For the visual TOJ task, we reported strong evidence in favor of the alternative hypothesis (BF_{10}= 14.092, error= 8.165e-7%) in the condition in which visual stimuli were presented in near space and the hands placed next to them. For the three other conditions we found moderate evidence in favor of the null hypothesis (visual stimuli in far space, hands close to near LEDs: BF_{10}= 0.483, error= 1.483e-4%; visual stimuli in near space, hands close to the body: BF_{10}= 0.795, error= 6.824e-5%; visual stimuli in far space, hands close to the body: BF_{10}= 0.279, error= 8.629e-5%)[2]. For the tactile TOJ task, we confirmed a lack of difference for both the uncrossed and the crossed hand posture, having a BF_{10} of 0.318 (2.131e-4% error) and 0.388 (2.131e-4% error), respectively, which can be interpreted as moderate-strong evidences in favor of the null hypothesis[2].

2.2 ANOVA with group factor

The ANOVAs of the TOJ parameters were initially performed by adding a group factor to assess whether the side of the affected hand (either left vs. right or dominant vs. non-dominant) could impact the other factors.

Analyses. The group factor was defined as either *affected hand* (left vs. right) or *dominance of the affected hand (*dominant vs. non-dominant*).* The ANOVAs for the visual TOJ tasks were therefore performed with *visual stimulus position*, *hand position* and one of the two group factors successively. Similarly, the ANOVAs for the tactile TOJ tasks were performed with *hand posture* and one of the group factors successively.

Results. Since the results were mostly the same whatever the added group factor, the reported results combine the two analyses and the values of the statistical tests relate to the analyses with the added factor *affected hand* and the factor *dominance of the affected hand,* respectively. Regarding the PSS values of the visual TOJ tasks, there was a significant main effect of *hand position* ($F(1,12)$= 5.153, p= .042, η^2p= .300; $F(1,12)$= 6.219, p= .028, η^2p= .341) and an effect of *visual stimuli position* that approached significance ($F(1,12)$= 4.081, p= .066, η^2p= .254; $F(1,12)$= 3.927, p= .071, η^2p= .247). The *hand position x visual stimuli position* interaction was not significant ($F(1,12)$= .013, p= .912, η^2p= .001; $F(1,12)$= .000, p= .995, η^2p= .000). There was no significant group difference ($F(1,12)$= .428, p= .525, η^2p= .034; $F(1,12)$= 1.073, p= .321, η^2p= .082), as well as no significant interaction with this factor (all $F \leq 1.674$, $p \geq .220$; all $F \leq 1.084$, $p \geq .318$). Regarding the slope values, the repeated measures ANOVAs did not show significant main effects or interactions (all $F \leq 1.167$, $p \geq 301$; all $F \leq 1.165$, $p \geq 302$), with an exception for the factor *hand position* which approached significance ($F(1,12)$= 4.565, p= .054, η^2p= .276) when using the *affected hand* group factor and which was significant when using the *dominance of the affected hand* group factor ($F(1,12)$= 5.446, p= .038, η^2p= .312). As for the PSS data, there was no significant difference between the groups ($F(1,12)$= .154, p= .701, η^2p= .013; $F(1,12)$= .000, p= .985, η^2p= .000),

nor any significant interaction with this factor (all $F \leq 3.313$, $p \geq .094$; all $F \leq 3.794$, $p \geq .075$). Regarding the PSS values for the tactile tasks, the ANOVAs did not show any significant difference, neither between the two conditions, nor between the groups, and no significant interaction between the factors (all $F \leq 1.281$, $p \geq .284$; all $F \leq 1.117$, $p \geq .315$). Regarding the slope values, none of the effects tested in the repeated measures ANOVA were significant (all $F \leq 1.415$, $p \geq .262$; all $F \leq 2.663$, $p \geq .134$).

3. References

1 Lewis, J. S. & Schweinhardt, P. Perceptions of the painful body: the relationship between body perception disturbance, pain and tactile discrimination in complex regional pain syndrome. *Eur J Pain* **16,** 1320-1330, 10.1002/j.1532-2149.2012.00120.x (2012).

2 Masson, M.E.J. A tutorial on a practical Bayesian alternative to null-hypothesis significance testing. *Behav Res* **43**, 679-690 (2011).

Supplementary table S1

Number of fulfilled criteria per symptom category and per sign category according to the Budapest criteria for all participants

	Reported Symptoms				Signs			
ID	Sensory	Vasomotor	Sudomotor/Edema	Motor/Trophic	Sensory	Vasomotor	Sudomotor/Edema	Motor/Trophic
01	n/a	n/a	n/a	n/a	n/a	n/a	n/a	n/a
02	1/1	1/2 t°	1/2 E	1/3 range	1/2 H	1/2 color	1/2 E	2/3 motor+range
03	n/a	n/a	n/a	n/a	n/a	n/a	n/a	n/a
04	n/a	n/a	n/a	n/a	n/a	n/a	n/a	n/a
05	n/a	n/a	n/a	n/a	n/a	n/a	n/a	n/a
06	1/1	2/2	0/2	2/3 troph+motor	0/2	1/2 color	0/2	2/3 troph+motor
07	0/1	2/2	2/2	3/3	1/2 H	1/2 color	1/2 E	2/3 troph+range
08	1/1	0/2	1/2 E	2/3 motor+range	1/2 A	0/2	0/2	2/3 motor+range
09	0/1	1/2 t°	2/2	3/3	1/2 A	1/2 t°	1/2 E	3/3
10	1/1	2/2	1/2 E	2/3 motor+range	2/2	2/2	1/2 E	2/3 motor+range
11	1/1	2/2	1/2 E	3/3	2/2	0/2	1/2 E	3/3
12	1/1	2/2	2/2	2/3 motor+range	2/2	2/2	2/2	2/3 motor+range
13	0/1	1/2 color	2/2	3/3	0/2	1/2 color	1/2 E	3/3
14	1/1	2/2	1/2 E	3/3	1/2 A	1/2 color	1/2 E	2/2 motor+range
15	1/1	2/2	2/2	3/3	1/2 A	1/2 t°	2/2	3/3
16	1/1	1/2 t°	0/2	2/3 motor+range	2/2	0/2	0/2	2/3 motor+range

Note. n/a= data not available; t°= temperature asymmetry; color= skin color changes; E= edema, range= decreased range of motion; troph= trophic changes; motor= motor dysfunction; H= hyperalgesia; A= allodynia.

Supplementary table S2

Additional clinical measures

ID	$Pain^{2days}$	T° affected (C°)	T° unaffected (C°)	Volume affected (ml)	Volume unaffected (ml)	DASH score	Activity affected-unaffected
01	4	31.7	30.3	164.7	185	30.8	n/a
02	3	24.5	26.5	156.6	151.3	54.2	n/a
03	5	31.2	32.6	328.3	345	60.8	n/a
04	5	33.2	33.5	156	165	47.5	n/a
05	5	24.6	25.8	121.6	126.6	40.8	-858.4
06	2	29.2	29.4	170	156.6	15.8	-816
07	1	28.2	27.2	318.3	278.3	54.1	-201.7
08	4	32	33.2	151.3	150	52.5	-83.1
09	4	33.1	34.5	148.3	160	60.8	n/a
10	4	29.6	30.9	255	191.6	59.2	n/a
11	2	33.5	33	191.6	146.6	60.8	n/a
12	7	29.4	30	383.3	363.3	63.3	n/a
13	7	35.6	34.3	216.6	225	81.7	-702.5
14	4	28.3	31.2	303.3	291.6	39.1	-1359.6
15	8	34.4	34.5	121.6	140	69.2	n/a
16	8	30.8	30.9	186.6	173.3	44.1	113.7

Note. $Pain^{2days}$ = average pain over the last 2 days rated on a visually presented numeric rating scale ranging from 0-10; T° affected= temperature of the affected hand; T° unaffected= temperature of the unaffected hand; Volume affected= volume of the affected hand, Volume unaffected= volume of the unaffected hand; DASH score= score calculated from the Disabilities of the Arm, Shoulder and Hand (DASH) questionnaire, smaller score= less impairment; n/a= data not available; Activity affected-unaffected= average vector magnitude count of the affected minus the unaffected hand, as assessed with accelerometers.
